# Public Awareness, Knowledge of Availability, And Willingness to Use Neurosurgical Care Services in Africa: A Cross-Sectional E-Survey Protocol

**DOI:** 10.29337/ijsp.149

**Published:** 2021-07-13

**Authors:** Chibuikem Ikwuegbuenyi, Gideon Adegboyega, Arsene Daniel Nyalundja, Michael A. Bamimore, Daniel Safari Nteranya, Lorraine Arabang Sebopelo, Ulrick Sidney Kanmounye

**Affiliations:** 1 Research Department, Association of Future African Neurosurgeons, Yaounde, Cameroon; 2 Queen Mary University of London, Barts and The London School of Medicine London, United Kingdom; 3 Department of Surgery, Hôpital Provincial General de Reference de Bukavu, Bukavu, Democratic Republic of Congo; 4 Faculty of Medicine, Université Catholique de Bukavu, Bukavu, Democratic Republic of Congo; 5 School of Medicine, Philadelphia College of Osteopathic Medicine, Philadelphia, PA, United States; 6 Department of Surgery, University Clinics of Bukavu, Official University of Bukavu, Bukavu, Democratic Republic of Congo; 7 Faculty of Medicine, University of Botswana, Gaborone, Botswana

**Keywords:** Africa, Health seeking behavior, Global neurosurgery

## Abstract

**Background:**

Barriers to care cause delays in seeking, reaching, and getting care. These delays affect low-and middle-income countries (LMICs), where 9 out of 10 LMIC inhabitants have no access to basic surgical care. Knowledge of healthcare utilization behavior within underserved communities is useful when developing and implementing health policies. Little is known about the neurosurgical health-seeking behavior of African adults. This study evaluates public awareness, knowledge of availability, and readiness for neurosurgical care services amongst African adults.

**Methodology:**

The cross-sectional study will be run using a self-administered e-survey hosted on Google Forms (Google, CA, USA) disseminated from 10th May 2021 to 10th June 2021. The Questionnaire would be in two languages, English and French. The survey will contain closed-ended, open-ended, and Likert Scale questions. The structured questionnaire will have four sections with 42 questions; Sociodemographic characteristics, Definition of neurosurgery care, Knowledge of neurosurgical diseases, practice and availability, and Common beliefs about neurosurgical care. All consenting adult Africans will be eligible. A minimum sample size of 424 will be used. Data will be analyzed using SPSS version 26 (IBM, WA, USA). Odds ratios and their 95% confidence intervals, Chi-Square test, and ANOVA will be used to test for associations between independent and dependent variables. A P-value <0.05 will be considered statistically significant. Also, a multinomial regression model will be used.

**Dissemination:**

The study findings will be published in an academic peer-reviewed journal, and the abstract will be presented at an international conference.

**Highlights:**

- The burden of neurosurgical diseases is enormous in low- and middle-income countries, especially in Africa.
- Unfortunately, most neurosurgical needs in Africa are unmet because of delays in seeking, reaching, and getting care.
- Most efforts aimed at reducing barriers to care have focused on improving the neurosurgical workforce density and infrastructure. Little or no efforts have been directed towards understanding or reducing the barriers to seeking care.
- We aimed to understand public awareness, willingness to use, and knowledge of the availability of neurosurgical care in Africa.
- The study findings can inform effective strategies that promote the utilization of neurosurgical services and patient education in Africa.

## Introduction

More than two-thirds of the global population lacks access to appropriate surgical care. An estimated 140 million necessary surgical procedures are left undone, resulting in excessive economic costs and profound disability and death [1]. There is a gross discrepancy in the availability of health resources for surgery, leaving a huge treatment gap geographically; this gap is seen particularly in Africa and Southeast Asia [2]. A huge disproportion exists in health care access in low- and middle-income countries (LMICs), where 9 out of 10 people have no access to basic surgical care [3]. There exist multiple factors that increase the delay to surgical care [4,5]. A widely used framework in many areas of global health, including surgery, is the three-delay framework – seeking, reaching, and receiving health care [6,7].

A review by Grimes et al. mentioned that barriers that limit access to surgery in LMICs are cultural (acceptability) and structural (accessibility), and to a significant extent, financial (affordability) [8]. Other studies identified low levels of health literacy and outdated medical facilities as barriers to surgical care [8,9,10,11,12,13,14,15]. Most of the efforts to reduce the barriers to neurosurgical care have been focused on improving the workforce density and the infrastructure via the provision of standard neurosurgical education and training and the provision of neurosurgical infrastructure [16,17]. While this is important, patients who need the services will only use them if they are willing to and are aware that these services exist in their community.

Healthcare-seeking behavior (HSB) is “any action or inaction which is undertaken by individuals who perceive themselves to have a health problem or to be ill to find an appropriate remedy” [18]. HSB can be appropriate or inappropriate. Appropriate HSB refers to “consulting a qualified medical professional or seeking healthcare at orthodox health facilities such as private clinics, primary health centers, and general hospitals during illness episodes or any situation requiring medical attention” [18]. Inappropriate HSB has been linked to worse health outcomes, increased morbidity and mortality, and poorer health statistics [19,20]. In Nigeria, rural dwellers are more likely to exhibit inappropriate HSB than urban dwellers [21]. Other determinants of HSB include geographical proximity, socio-economic class, culture, political affiliation, education level, and the healthcare systems themselves [22,23]. Most rural dwellers use health facilities outside their villages because they lack access to these services in their local areas [24].

Africans attribute metaphysical characteristics, such as witchcraft/sorcery, to neurological diseases, including epilepsy, and this, in turn, causes stigmatization of people with epilepsy, thereby affecting the HSB [24]. In Ethiopia, patients report that their religion plays a critical role in their attitude towards neurosurgical procedures [25]. Similarly, Nigerian patients turn to religion for comfort after learning about their neurosurgical disease [26]. In Kenya, almost 70% of pregnant women within households in the upper socio-economic stratum deliver in health facilities compared with 42% among pregnant women in the middle socio-economic stratum and 38% in the low socio-economic stratum [27].

The lack of knowledge and general misperceptions regarding neurosurgical care are considerable barriers to neurosurgical care, and further education of the general public would be of great value. To develop effective strategies to promote the utilization of neurosurgical services in Africa, we must explore demographic characteristics and barriers related to general public awareness of neurosurgical services and intentions for use and knowledge of availability. Knowledge of healthcare utilization behavior within communities is useful when developing health policies and implementing health programs [28]. We, therefore, aim with this paper to understand the public awareness of neurosurgical care, willingness to use, and knowledge of the availability.

### 1.1. Aims and Objectives

**Aims:** To map out public awareness, knowledge of availability, and readiness for neurosurgical care services amongst adult Africans.

## 2. Methods and Analysis

### 2.1. Study Design

This cross-sectional study will use a self-administered e-survey to collect public awareness, knowledge of availability, and readiness for neurosurgical care services in Africa. A team of African neurosurgeons and patients will be consulted to assess the questionnaire’s face validity. This team will check the questionnaire for duplication, confusing terms, and leading questions. We will use the team’s feedback to improve the survey. Next, the questionnaire will be piloted among an arbitrary sample size of 20 respondents to identify technical issues with the survey. We will use Cronbach’s alpha to evaluate the questionnaire’s internal consistency and principal component analysis to identify factor loadings. Next, we will make revisions accordingly.

### 2.2. Eligibility Criteria

- **Inclusion Criteria:** All consenting adult Africans who are not studying towards a healthcare degree or working in the healthcare sector.
- **Exclusion Criteria:** All medical/dental/nursing/allied health students and workers. This group will be excluded because it is expected that members will have better knowledge and more positive attitudes towards neurosurgical care than the general public.

### 2.3. Data Collection Tools and Technique

Self-administered structured Google Form (Google, CA, USA) questionnaires in French and English will be used for the study. The survey will contain closed-ended, open-ended, and Likert Scale questions. The structured questionnaire will have four sections (A–D). They are:

Section A – Sociodemographic characteristics (7): Age, gender, marital status, Nationality, Occupation, Urban/Rural settlement, Length of living in the region.

Section B – Definition of neurosurgery care (2): Respondents will be asked to define neurosurgery in their own words, then asked to select from several options disorders that can be treated with Neurosurgical care.

Section C – Knowledge of neurosurgical diseases, practice, and availability (11).

Section D – Common beliefs about neurosurgical care (12).

Written consent will be obtained from the respondents before the administration of questionnaires.

### 2.4. Sample Size

The sample size was determined using the Normal approximation to the hypergeometric: n = n = Nz^2pq/(E^2(N–1) + z^2pq) [29]

Where;

n = Minimum required sample size

N = the population size (1,362,565,076) [30]

z = Confidence level at 95 % = 1.96

P and q are the population proportions. (These were set to 0.5)

d = Margin of error (5% = 0.05)

Employing a standard normal deviate of 1.96% at 95% confidence level and a maximal allowable difference from a true population of 5% (0.05), the sample size will be:

n = Nz^2pq/(E^2(N–1) + z^2pq)

    = 385

The sample size was then increased by 10% to allow for contingencies such as non-response.

n + 10% of n = 385 + [(10/100) * 385]

                      = 385 + (0.1 * 385)

                      = 382 + 38.5

                      = 423.5

                      = 424

Therefore, the sample size (n) is equal to 424.

### 2.5 Outcome Measures

The primary outcome is the level of knowledge about the availability of the services.

Secondary outcomes will include the level of knowledge and beliefs about neurosurgical disease and the knowledge level of neurosurgical practice in Africa.

### 2.6. Data Analysis

Independent variables will include age, sex, marital status, profession, nationality, urban/rural residency, previous experience with neurosurgery. Dependent variables will include the level of knowledge and willingness to use neurosurgical services.

Data will be analyzed using SPSS version 26 (IBM, WA, USA). The analyzed univariable data will be presented as frequency tables and charts. The levels of knowledge will be stratified into five groups equally spaced: deficient, sufficient, satisfactory, good, and very good. The Q-Q plot will be used to evaluate the distribution of quantitative data. Also, the odds ratios and their 95% confidence intervals, Chi-Square or Fisher’s Exact Test, and ANOVA or Kruskal-Wallis test will be used to quantify associations between independent and dependent variables. Next, the independent variables will be integrated into a multinomial regression model.

### 2.7 Ethics

Approval to carry out this study was obtained from the institutional review board of Bel Campus University of Technology, Kinshasa, DRC (No 2135488). The participants will be informed of the reason and nature of the study. Informed consent will be sought, and the participants will be informed of their right to withdraw at any point of the study. Participants will not be coerced to participate in the study, and confidentiality will be maintained throughout the study.

## 3. Limitations

Internet access remains low in many African countries, making this study inaccessible to a wide audience, especially those who seek or have used neurosurgical facilities in remote areas. Therefore, this provides an opportunity for researchers to conduct a large population-based survey in the future to get an idea of the ampleness of public awareness, knowledge of availability, and availability of neurosurgical care services in Africa by including both the population in remote areas and those who do not speak English or French.

Socioeconomic status and the education level of citizens are some of the factors that will limit the generalizability of our results. Citizens in the higher income bracket and those with a higher level of education are perhaps more likely to have more access to the internet and a better understanding of research goals.

## 4. Conclusion

Although there has been an increase in access to neurosurgical care in Africa, the unmet need remains significant. One reason this might be the case is that African neurosurgical patients experience multiple barriers to seeking care. Therefore, we must understand the role of HSBs in bridging the treatment gap. This study will survey the public awareness of neurosurgical care services intended for use, knowledge of availability, and readiness for neurosurgical care services in Africa.

## 5. Dissemination

We plan to publish this review in a peer-reviewed journal. We may also present this review at local and/or international conferences.

## Additional Files

The additional file for this article can be found as follows:

10.29337/ijsp.149.s1 Appendix 1. Questionnaires in French and English, Adapted from [31].

## Abbreviations

LMICs, Low-and middle-income countries; HSB, Healthcare seeking behavior; MT, Medical tourism.
