# Supplementary material for: Public Awareness, Knowledge of Availability, And Willingness to Use Neurosurgical Care Services in Africa: A Cross-Sectional E-Survey Protocol
Source: Int J Surg Protoc. 2021 Jul 13;25(1):123–8. doi: 10.29337/ijsp.149 (PMC8284504; doi:10.29337/ijsp.149)
Supplement: Appendix 1. — Questionnaires in French and English, Adapted from [31]. [file ijsp-25-1-149-s1.zip › French Survey.pdf]

Sensibilisation du public, connaissance de la disponibilité et de la disponibilité des services de soins neurochirurgicaux en Afrique

Informations générales

L'objectif de cette étude est de recueillir des données pertinentes et de haute qualité concernant la connaissance, la disponibilité et la disponibilité des services de neurochirurgie.

Nous apprécions votre intérêt à participer à ce questionnaire. Vous avez été invité(e) à participer car vous êtes âgé(e) de 18 ans ou plus, et résidez en Afrique. Veuillez lire ces informations avant d'accepter de participer en cochant la case "oui" ci-dessous.

Il vous sera demandé de répondre à des questions concernant la connaissance, la disponibilité et la disponibilité des services de neurochirurgie. Cela devrait prendre moins de 10 minutes. Aucune connaissance de base n'est requise. Les données recueillies seront utilisées à des fins de recherche, elles seront stockées de manière sécurisée et ne seront accessibles qu'aux chercheurs principaux et aux utilisateurs qu'ils auront désignés. À la fin du projet, les données seront conservées pendant cinq ans après la publication finale.

Suis-je obligé de participer ?

Veuillez noter que votre participation est volontaire. Si vous décidez de participer, vous pouvez vous retirer à tout moment du questionnaire pour n'importe quelle raison avant de soumettre vos réponses en appuyant sur la touche de fermeture du navigateur.

Comment mes données seront-elles utilisées ?

Vos réponses seront totalement anonymes et nous prendrons toutes les mesures raisonnables pour en assurer la confidentialité.

Vos données seront stockées dans un fichier protégé par un mot de passe et pourront être utilisées dans des publications universitaires. Votre adresse IP ne sera pas conservée. Toutes les questions sont facultatives. Les données de recherche seront conservées pendant au moins trois ans après leur publication ou leur diffusion publique. Les données que nous recueillons auprès de vous peuvent être transférées, stockées et/ou traitées vers une destination située en dehors de votre pays et de votre continent. En soumettant vos données personnelles, vous acceptez ce transfert, ce stockage ou ce traitement.

Qui aura accès à mes données ?

Google est le responsable du traitement de vos données personnelles et, en tant que tel, il

déterminera l'utilisation de vos données personnelles. Veuillez consulter leur avis de confidentialité ici https://policies.google.com/privacy?hl=en-US. Google ne partagera que des données entièrement anonymes avec tous les membres de l'équipe de recherche aux fins de cette étude.

Nous souhaitons également obtenir votre autorisation d'utiliser vos données anonymes dans le cadre d'études futures et de les partager avec d'autres chercheurs (par exemple, dans des bases de données en ligne). Toute information personnelle permettant de vous identifier sera supprimée ou modifiée avant que les fichiers ne soient partagés avec d'autres chercheurs ou que les résultats ne soient rendus publics.

Qui dois-je contacter si j'ai un problème avec l'étude ou si je souhaite déposer une plainte ?

Si vous avez un souci concernant un aspect quelconque de cette étude, veuillez contacter le chercheur principal à l'adresse ikwuegbuenyichibuikem@gmail.com.

* Required

1. Email address *

____

Skip to question 2Skip to question 2

Formulaire de consentement

En cliquant sur le bouton ci-dessous, vous reconnaissez que votre participation à l'étude est volontaire, que vous avez au moins 18 ans et que vous êtes conscient que vous pouvez choisir de mettre fin à votre participation à l'étude à tout moment et pour n'importe quelle raison.

2. Veuillez indiquer votre consentement avant de poursuivre *

Mark only one oval.

- ⬭ Je consens *Skip to question 3*
- ⬭ Je ne consens pas *Skip to section 8 (Terminé)*

Critères d'éligibilité

3. Êtes-vous un professionnel de la santé (médecin, infirmier ou professionnel paramédical) ou un étudiant dans une profession de la santé ? *

Mark only one oval.

- Oui *Skip to section 8 (Terminé)*
- Non *Skip to question 4*

Caractéristiques sociodémographiques

4. Âge (années) *

Mark only one oval.

- 18
- 19
- 20
- 21
- 22
- 23
- 24
- 25
- 26
- 27
- 28
- 29
- 30
- 31
- 32
- 33
- 34
- 35
- 36
- 37
- 38
- 39
- 40
- 41+

5. Sex *

Mark only one oval.

- Female
- Male

6. État civil *

Mark only one oval.

- Marié(e)
- Célibataire
- Divorcé(e)
- Veuf(ve)

7. Occupation/Profession *

____

8. Pays *

Mark only one oval.

- Algeria
- Angola
- Benin
- Botswana
- Burkina Faso
- Burundi
- Cameroon
- Cabo Verde
- Central African Republic
- Chad
- Comoros
- Congo, The Democratic Republic
- Congo, The Republic
- Côte d'Ivoire
- Djibouti
- Equatorial Guinea
- Egypt
- Eritrea
- Ethiopia
- Gabon
- Gambia
- Ghana
- Guinea
- Guinea-Bissau
- Kenya
- Lesotho, The Kingdom of
- Liberia
- Libya
- Madagascar
- Malawi

- [illegible]
- Mali
- Mauritania
- Mauritius
- Morocco
- Mozambique
- Namibia
- Niger
- Nigeria
- Rwanda
- Sao Tome and Principe
- Senegal
- Seychelles
- Sierra Leone
- Somalia
- South Africa
- South Sudan
- Sudan
- Swaziland, Kingdom of
- Tanzania
- Togo
- Tunisia
- Uganda
- Zambia
- Zimbabwe

9. Vous vivez dans une zone *

Mark only one oval.

- Urbaine
- Rurale

10. Depuis combien de temps vivez-vous dans la région ? years=année) *

Mark only one oval.

- 1
- 2
- 3
- 4
- 5
- 6
- 7
- 8
- 9
- 10
- 11
- 12
- 13
- 14
- 15
- 16
- 17
- 18
- 19
- 20
- 21
- 22
- 23
- 24
- 25
- 26
- 27
- 28
- 29

- 30
- 31 ou plus

Définition

11. Veuillez définir la neurochirurgie ou la chirurgie neurologique dans vos propres mots ? *

____
____
____
____
____

12. Parmi les maladies suivantes, lesquelles peuvent être traitées par un neurochirurgien/chirurgien neurologue ? *

Check all that apply.

- [ ] Accident vasculaire cérébral ou accident cérébrovasculaire
- [ ] Cancers de la colonne vertébrale et de la moelle épinière (ex : métastase, épendymome, méningiome)
- [ ] Lésion traumatique du cerveau/de la tête (ex : hématome épidural, hématome sous-dural)
- [ ] Maladies du rein (ex : pyélonéphrite, cancer du rein)
- [ ] Lésion traumatique de la colonne vertébrale (ex : lésion de la moelle épinière, hématome épidural)
- [ ] Maladies de la prostate (ex : hyperplasie bénigne de la prostate, cancer de la prostate)
- [ ] Épilepsie (ex : épilepsie qui ne peut être traitée par des médicaments)
- [ ] Cancers du cerveau (ex : méningiome, gliome, métastases)
- [ ] Compression des nerfs (ex : sciatique)
- [ ] Malformations du cerveau et de la colonne vertébrale (ex : hydrocéphalie, spina bifida)

Connaissance des maladies, de la pratique et de la disponibilité de la neurochirurgie.

"La neurochirurgie ou chirurgie neurologique est la spécialité médicale qui s'occupe de la prévention, du diagnostic, du traitement chirurgical et de la réadaptation des troubles qui affectent toute partie du système nerveux, notamment le cerveau, la moelle épinière, le système nerveux central et périphérique, et le système cérébro-vasculaire." "Description de la spécialité de la chirurgie neurologique". Association médicale américaine. Consulté le 4 octobre 2020.

13. Subissez-vous ou avez-vous subi un traitement neurochirurgical ? *

Mark only one oval.

- Oui
- Non

14. Avez-vous ou avez-vous eu des membres de votre famille qui ont subi un traitement neurochirurgical ? *

Mark only one oval.

- Oui
- Non

15. Connaissez-vous ou avez-vous entendu parler des neurochirurgiens dans votre pays ? *

Mark only one oval.

- Oui
- Non

16. S'il existe des neurochirurgiens dans votre pays, savez-vous dans quels hôpitaux ils travaillent ?

Mark only one oval.

- Oui
- Non

17. Si vous ou un de vos proches avait besoin de soins neurochirurgicaux, utiliseriez-vous les services de votre pays ? *

Mark only one oval.

- Oui
- Non

18. Pourquoi ? *

19. Parmi ces pays africains, quels sont ceux qui, à votre connaissance, disposent de services de neurochirurgie ? *

Check all that apply.

- [ ] Algeria
- [ ] Angola
- [ ] Benin
- [ ] Botswana
- [ ] Burkina Faso
- [ ] Burundi
- [ ] Cameroon
- [ ] Cabo Verde
- [ ] Central African Republic
- [ ] Chad
- [ ] Comoros
- [ ] Congo, The Democratic Republic
- [ ] Congo, The Republic
- [ ] Côte d'Ivoire
- [ ] Djibouti
- [ ] Equatorial Guinea
- [ ] Egypt
- [ ] Eritrea
- [ ] Ethiopia
- [ ] Gabon
- [ ] Gambia
- [ ] Ghana
- [ ] Guinea
- [ ] Guinea-Bissau
- [ ] Kenya
- [ ] Lesotho, The Kingdom of
- [ ] Liberia
- [ ] Libya
- [ ] Madagascar
- [ ] Malawi
- [ ] Mali
- [ ] Mauritania
- [ ] Mauritius
- [ ] Morocco
- [ ] Mozambique

- [ ] Namibia
- [ ] Niger
- [ ] Nigeria
- [ ] Rwanda
- [ ] Sao Tome and Principe
- [ ] Senegal
- [ ] Seychelles
- [ ] Sierra Leone
- [ ] Somalia
- [ ] South Africa
- [ ] South Sudan
- [ ] Sudan
- [ ] Swaziland, Kingdom of
- [ ] Tanzania
- [ ] Togo
- [ ] Tunisia
- [ ] Uganda
- [ ] Zambia
- [ ] Zimbabwe

20. Si vous ou l'un de vos proches aviez besoin de soins neurochirurgicaux, utiliseriez-vous les services d'un autre pays africain ? *

Mark only one oval.

( ) Oui

( ) Non

21. Pourquoi ? *

22. Si vous êtes prêt à utiliser les services neurochirurgicaux d'un autre pays africain, dans lequel de ces pays iriez-vous ?

Check all that apply.

- [ ] Algeria
- [ ] Angola
- [ ] Benin
- [ ] Botswana
- [ ] Burkina Faso
- [ ] Burundi
- [ ] Cameroon
- [ ] Cabo Verde
- [ ] Central African Republic
- [ ] Chad
- [ ] Comoros
- [ ] Congo, The Democratic Republic
- [ ] Congo, The Republic
- [ ] Côte d'Ivoire
- [ ] Djibouti
- [ ] Equatorial Guinea
- [ ] Egypt
- [ ] Eritrea
- [ ] Ethiopia
- [ ] Gabon
- [ ] Gambia
- [ ] Ghana
- [ ] Guinea
- [ ] Guinea-Bissau
- [ ] Kenya
- [ ] Lesotho, The Kingdom of
- [ ] Liberia
- [ ] Libya
- [ ] Madagascar
- [ ] Malawi
- [ ] Mali
- [ ] Mauritania
- [ ] Mauritius
- [ ] Morocco
- [ ] Mozambique

- [ ] Namibia
- [ ] Niger
- [ ] Nigeria
- [ ] Rwanda
- [ ] Sao Tome and Principe
- [ ] Senegal
- [ ] Seychelles
- [ ] Sierra Leone
- [ ] Somalia
- [ ] South Africa
- [ ] South Sudan
- [ ] Sudan
- [ ] Swaziland, Kingdom of
- [ ] Tanzania
- [ ] Togo
- [ ] Tunisia
- [ ] Uganda
- [ ] Zambia
- [ ] Zimbabwe

23. Pourquoi ?

____
____
____
____
____

24. Si vous ou l'un de vos proches aviez besoin de soins neurochirurgicaux, utiliseriez-vous ces services dans un pays non africain ? *

Mark only one oval.

( ) Oui
( ) Non

25. Pourquoi ? *

26. Si vous êtes prêt à utiliser les services neurochirurgicaux d'un pays non africain, dans quelle région vous rendriez-vous ?

Mark only one oval.

- Australie
- Asie
- Europe
- Amérique centrale
- Amérique du nord
- Amérique du sud

27. Pourquoi ?

28. S'il n'y avait aucun obstacle, lequel des services neurochirurgicaux suivants utiliseriez-vous en premier ? *

Mark only one oval.

- Dans votre pays
- Dans un autre pays d'Afrique
- Dans un pays non-africain

29. Pourquoi ? *

Croyances communes sur les soins neurochirurgicaux

30. Vous ne devriez consulter un neurochirurgien qu'en dernier recours ou lorsqu'une intervention chirurgicale est nécessaire *

Mark only one oval.

- Pas du tout d'accord
- Pas d'accord
- Neutre
- D'accord
- Tout à fait d'accord

31. La neurochirurgie est coûteuse *

Mark only one oval.

- Pas du tout d'accord
- Pas d'accord
- Neutre
- D'accord
- Tout à fait d'accord

32. Si ma tête est opérée, je ne serai plus jamais le même. *

Mark only one oval.

- Pas du tout d'accord
- Pas d'accord
- Neutre
- D'accord
- Tout à fait d'accord

33. Je fais davantage confiance à mon/ma neurochirurgien(ne) s'il/elle a été formé(e) à l'étranger. *

Mark only one oval.

- Pas du tout d'accord
- Pas d'accord
- Neutre
- D'accord
- Tout à fait d'accord

34. Je fais davantage confiance à mon/ma neurochirurgien(ne) s'il/elle est plus âgé(e). *

Mark only one oval.

- Pas du tout d'accord
- Pas d'accord
- Neutre
- D'accord
- Tout à fait d'accord

35. Je fais davantage confiance à mon/ma neurochirurgien(ne) s'il/elle a été recommandé(e) par une personne que je connais. *

Mark only one oval.

- Pas du tout d'accord
- Pas d'accord
- Neutre
- D'accord
- Tout à fait d'accord

36. Je fais davantage confiance à mon/ma neurochirurgien(ne) s'il/elle a traité avec succès une célébrité (ex : politicien, musicien, personne fortunée). *

Mark only one oval.

- Pas du tout d'accord
- Pas d'accord
- Neutre
- D'accord
- Tout à fait d'accord

37. Je fais plus confiance à mon neurochirurgien s'il est un homme. *

Mark only one oval.

- Pas du tout d'accord
- Pas d'accord
- Neutre
- D'accord
- Tout à fait d'accord

38. Je fais plus confiance à mon neurochirurgien s'il est une femme. *

Mark only one oval.

- Pas du tout d'accord
- Pas d'accord
- Neutre
- D'accord
- Tout à fait d'accord

39. Je fais davantage confiance à mon/ma neurochirurgien(ne) s'il/elle collabore souvent avec des chirurgiens étrangers *

Mark only one oval.

- Pas du tout d'accord
- Pas d'accord
- Neutre
- D'accord
- Tout à fait d'accord

40. Les maladies neurochirurgicales peuvent être traitées par la médecine traditionnelle *

Mark only one oval.

- Pas du tout d'accord
- Pas d'accord
- Neutre
- D'accord
- Tout à fait d'accord

41. Les maladies neurochirurgicales peuvent être traitées grâce à l'intervention spirituelle des chefs religieux *

Mark only one oval.

- Pas du tout d'accord
- Pas d'accord
- Neutre
- D'accord
- Tout à fait d'accord

Terminé

Merci d'avoir répondu à cette enquête

This content is neither created nor endorsed by Google.

Google Forms
